# A Novel Self-Assembling Al-based Composite Powder with High Hydrogen Generation Efficiency

**DOI:** 10.1038/srep17428

**Published:** 2015-11-30

**Authors:** Cuiping Wang, Yuheng Liu, Hongxin Liu, Tao Yang, Xinren Chen, Shuiyuan Yang, Xingjun Liu

**Affiliations:** 1 Department of Materials Science and Engineering, College of Materials, Xiamen University, Xiamen, 361005, P. R. China; 2 Research Center of Materials Design and Applications, Xiamen University, Xiamen, 361005, P. R. China; 3 Collaborative Innovation Center of Chemistry for Energy Materials, Xiamen University, Xiamen 361005, P. R. China

## Abstract

In this study, a novel self-assembling hydrogen generation powder comprised of 80Al-10Bi-10Sn wt.% was prepared using the gas atomization method and then collected in an air environment. The morphological and hydrolysis properties of the powders were investigated. The results indicated that the powders formed unique core/shell microstructures with cracked surfaces and (Bi, Sn)-rich phases distributed on the Al grain boundaries. The powders exhibited good oxidation resistance and reacted violently with distilled water at temperatures as low as 0 °C. Furthermore, at 30 °C, the powders exhibited a hydrogen conversion yield of 91.30% within 16 minutes. The hydrogen produced by this powder could be directly used in proton exchange membrane fuel cells. The mechanisms of the hydrolysis reactions were also analyzed.

Due to increasing worldwide energy demands and environmental concerns, studies concerning the use of hydrogen (H_2_) as an energy source have become prevalent in the past few decades^1^,^2^,^3^,^4^,^5^,^6^. To date, aluminum (Al)-water reactions in neutral aqueous solutions have been widely recognized as a promising method of H_2_ generation due to its simplicity, safety, effectiveness, and environmental safety^7^,^8^,^9^. However, the efficiency of H_2_ generation is significantly limited by the formation of a dense passive oxide layer on the surface of aluminum and its alloys. Numerous attempts to remove this passive oxide layer and, thereby, improve the H_2_ generation efficiency of aluminum water reactions have been made^10^,^11^,^12^,^13^. Among these existing strategies, the use of the ball milling method to fabricate Al-based powders containing low-melting-point metals^14^,^15^,^16^, active metals^17^,^18^,^19^, and other additives^20^,^21^,^22^,^23^ has been considered to be the most effective way to facilitate the continuous generation of H_2_. Nevertheless, this method still suffers from several disadvantages such as high cost, low efficiency and environmental pollution. In addition, the powders fabricated with this method are not readily available, are unstable, and quickly lose their chemical functionality when exposed to air^7^,^14^. It is thus expected to remove the above limitations by introducing a protective surface layer to prevent the oxidation of Al while allowing it to react with water simultaneously.

In the previous studies of the present authors, self-assembling egg-type powders with core/shell microstructures were produced via gas atomization processing in the Iron (Fe)-Copper (Cu) liquid immiscible system^24^ Inspired by this, self-assembling powders with cores comprised of Al and shells comprised of other protective alloying elements in the Al-based liquid immiscible system are anticipated not only to avoid surface oxidation, but also to improve the activity of the Al-based materials for H_2_ generation.

Bi and Sn were selected as the alloying elements in this study due to the stable and the metastable liquid miscibility gaps in the Al-Bi^25^ system and Al-Sn^26^ systems, respectively. Composite powders comprised of 80Al-10Bi-10Sn (wt.%, and hereafter) were prepared through gas atomization processing and then collected in an air environment. The hydrogen generation properties and reaction mechanisms of this powder in distilled water were investigated.

## Results

### Surface morphologies and cross-sectional microstructures of the composite powders

**Figure 1:**
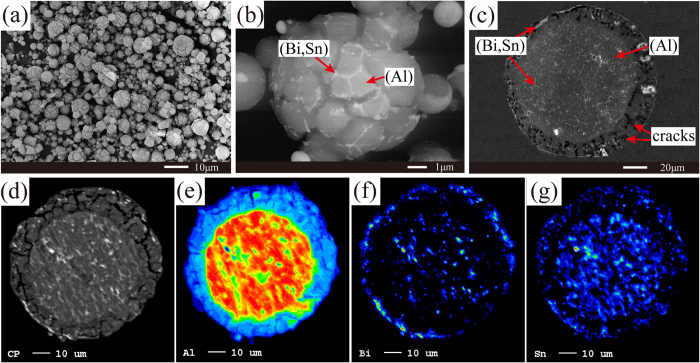
(**a–c**) SEM images of the 80Al-10Bi-10Sn wt.% powders: (**a,b**) surfaces, (**c**) cross-section; (**d–g**) EPMA element mappings of the cross section of the powders: (**d**) BSE, (**e**) Al, (**f**) Bi, and (**g**) Sn.

The morphologies of the 80Al-10Bi-10Sn powders are shown in Fig. 1. As shown in Fig. 1a,b, the microstructures of the 80Al-10Bi-10Sn powders were characterized by cracked surfaces with linear (Bi, Sn)-rich phases spreading throughout the grain boundaries. Moreover, as can be seen in the cross-section in Fig. 1c and the electron probe microanalysis (EPMA) element mappings in Fig. 1(d–g), the powders were enveloped by a thin, discontinuous (Bi, Sn)-rich shell. According to Liu^27^, the Al-Bi-Sn ternary system also exhibited a liquid-phase miscibility gap (Supplementary Figures S1a and S2). During the cooling process of gas atomization, for every droplets, the parent liquid phase decomposed into the minor (Bi, Sn)-rich phase and the major Al-rich phase^24^. According to our previous studies, the final morphologies of core/shell microstructures of the composite powders are determined by the differences of volume fractions and the differences of surface energies between the separated phases^28^,^29^. Under the fast cooling conditions of gas atomization, there exists a large temperature gradient in the droplets. Therefore, the influences of the differences of surface energies play a greater role in this case^24^. In order to reduce the energy of the whole powder, the (Bi, Sn)-rich phase with lower surface energies moved outward, wetting and occupying the powder surface. However, the volume of the (Bi, Sn)-rich phase was too small to entirely cover the powder surface (Supplementary Figure S1b). In addition, since the composition of the (Bi, Sn)-rich phase was very close to the eutectic composition of the Bi-Sn system^27^, its solidifying point was much lower than that of the Al-rich phase. As the temperature decreased, the Al-rich phase firstly solidified, while the (Bi, Sn)-rich phase remained in the liquid state and was homogeneously distributed around the grain boundaries of the outer layers of the powders. The cracked surfaces were attributed to the differences in the thermal expansion coefficients of the Al, Sn, and Bi^30^ (23.0 ppm k^−1^, 21.9 ppm k^−1^, and 13.2 ppm k^−1^ at 20 °C, respectively). Under the fast-cooling conditions of gas atomization, the Al-rich and (Bi, Sn)-rich phases on the grain boundaries shrank for different degrees, leading to the crack of the powder surface. As for the small (Bi, Sn)-rich phases distributed on the grain boundaries inside the powders, as shown in Fig. 1c, they were supersaturated from the Al-rich phase when the temperature decreased. In order to prove our assumption for the crack of the powder surfaces, powders comprised of 85Al-8.55Bi-6.45Sn wt.%, in which the compositions of Bi and Sn were located just on the eutectic one of the Bi-Sn system, were prepared using the same method. The results show that, the surfaces of the powders cracked more heavily (Supplementary Figure S3).

### Hydrogen generation properties of the composite powders

**Figure 2:**
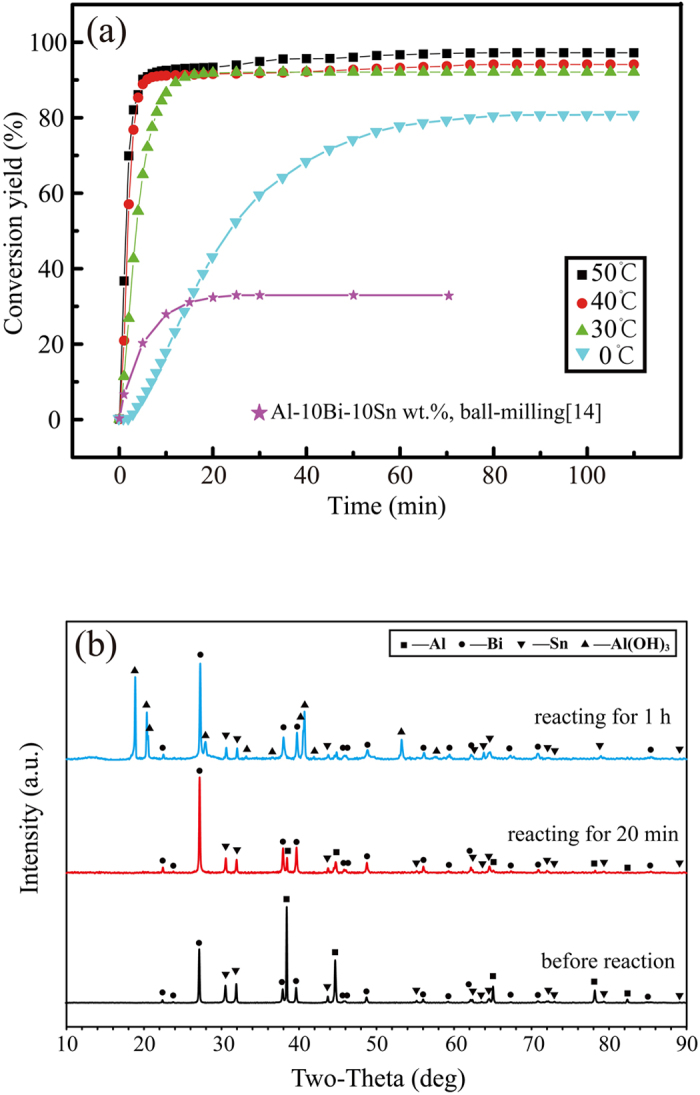
(**a**) Hydrogen generation curves of the 80Al-10Bi-10Sn wt.% powders in distilled water; (**b**) XRD patterns of the powders after reacting with the distilled water at 30 °C for different amounts of time.

The hydrogen generation properties of the composite powders in distilled water were investigated and the results are shown in Fig. 2a. The 80Al-10Bi-10Sn powders exhibited superior hydrolysis characteristics and reacted immediately with distilled water. At 30 °C, the reactions occurred rapidly and the conversion yield increased to 91.30% within 16 minutes. Higher temperatures promoted the reactions, the reactions at 40 °C and 50 °C achieved higher conversion yields in smaller amounts of time. Even at 0 °C, the reactions were still violent, achieving a conversion yield of 80.43% after 88 minutes. The final conversion yield of the reactions at 0 °C, 30 °C, 40 °C and 50 °C is 80.56%, 92.02%, 94.05% and 97.15%, respectively. The reason for the conversion yield not reaching 100% is the unavoidable oxidation of the bare Al-phase on the powder surface during the powder collection process in air environment. Thus, a little amount of Al is consumed before the hydrogen generation experiments (Supplementary Figure S4). It is worth noting that, the conversion curve labeled with a pentagram in Fig. 2a represents the conversion curve of 80Al-10Bi-10Sn wt.% materials (with the same composition as that of the composite powders used in this study) prepared through the ball milling method in pure water at room temperature^14^. It is obvious that, although the self-assembling powders in this study were collected in an air environment, they exhibited relatively high hydrogen generation rates and conversion yields, indicating the composite powders in this study had good oxidation resistance and higher hydrogen generation efficiency. Prolonging the exposure time in air environment, the conversion yield of the composite powders almost kept the same value (Supplementary Figure S5).

In addition, the hydrogen produced by the composite powders in this study could be used directly in proton exchange membrane fuel cells (PEMFC), through which the LED light, fan, and mobile phone could be charged using a USB interface (Supplementary Figure S6 and Video S1).

Figure 2b displays the XRD patterns of the 80Al-10Bi-10Sn powders after reacting with distilled water at 30 °C for different amounts of time. As shown in Fig. 2b, before the reactions occurred, only the diffraction peaks of Al, Bi, and Sn were visible. As the reaction time increased, the Al diffraction peaks decreased, and the Al(OH)_3_ diffraction peaks increased. In addition, the Bi and Sn diffraction peaks did not exhibit any significant changes, indicating that only the Al reacted with the water.

### Microstructures and mechanisms of the powders during hydrolysis reactions

**Figure 3:**
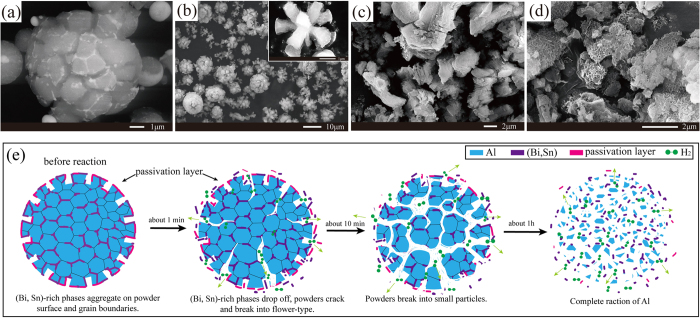
(**a–d**) SEM images of the 80Al-10Bi-10Sn wt.% powders reacting with distilled water at 30 °C for different amounts of time: (**a**) before the reactions, (**b**) after 30 seconds, (**c**) after 10 minutes, and (**d**) after 1 hour; (**e**) schematic illustration of the reaction mechanisms of the powders reacting with distilled water at 30 °C.

In order to investigate the reaction mechanisms of the powders, the solid hydrolysis products of the powders in distilled water at 30 °C for different amounts of time were analyzed. The results are shown in Fig. 3(a–d). As Fig. 3a shows, before the reactions occurred, the powders were characterized by cracked surfaces with linear (Bi, Sn)-rich phases throughout the grain boundaries. After reacting with distilled water for only 30 seconds (Fig. 3b), the powders became detached from the grain boundaries, forming flower-like structures. After 10 minutes, the powders were broken into small particles (Fig. 3c). After 1 hour, only latticed Al(OH)_3_ was visible (Fig. 3d)^31^,^32^,^33^.

Figure 3e is the schematic diagram of the hydrolysis reactions of the powders in distilled water at 30 °C. Before the reactions occurred, the powder surfaces were enveloped by a thin, discontinuous (Bi, Sn)-rich shell; while the bare Al phases were covered by passivation layers, which were formed during the collection process in the air environment. However, the cracked powder surfaces and the (Bi, Sn)-rich phases spreading equally on the grain boundaries broke the dense oxide layers on the surfaces of Al. Besides, the cracked powder surfaces increased the amount of surface area available for hydrolysis reactions. As known to all, the hydrolysis reaction of Al in water is exothermal^7^. When in contact with water, the (Bi, Sn)-rich phases on the powder surfaces, especially on the grain boundaries, were easily removed due to the large differences in their thermal expansion coefficients (Supplementary Figures S7 and S8). As a result, fresh surfaces of Al were exposed to water, from where violent hydrolysis reactions started. Before long, the powders became detached from the grain boundaries and formed flower-like structures because of the consumption of Al and the expansion of the hydrogen bubbles produced by the hydrolysis reaction. Thus, more and more Al grains with fresh surface were exposed to water, resulting in violent hydrolysis reactions and highly efficient of hydrogen generation.

## Discussion

In this study, highly active Al-based self-assembling composite powders with novel structures were successfully prepared using the gas atomization method. The hydrogen generation characteristics and reaction mechanisms of the composite powders in distilled water were investigated. Under fast-cooling conditions of gas atomization, the 80Al-10Bi-10Sn powders form unique core/shell structures with cracked surfaces and (Bi, Sn)-rich phases distributed on the grain boundaries. These characteristics are attributed to the liquid phase miscibility gap, the differences of surface energies of the separated liquid phases and the differences in the thermal expansion coefficients of Al, Bi, and Sn.

The (Bi, Sn)-rich phase plays two important roles in improving the H_2_ generation efficiency: (a) the (Bi, Sn)-rich phase forms an incomplete shell covering the powder surface, preventing the Al in the core from oxidation by oxygen; (b) (Bi, Sn)-rich phase distributes on the grain boundaries of the Al phases, together with the cracks on powder surfaces, they break the dense passivation layers on the surfaces of Al. When contacting with water, the cracks increase the specific surface area of the powders. More important, both the (Bi, Sn)-rich phases on the powder surface and on the grain boundaries can drop off the powders easily due to the large differences of thermal expansion coefficients between (Bi, Sn)-rich phase and Al, which is beneficial for the contacting between water and fresh Al surface, resulting in high H_2_ generation efficiency and favorable oxidation resistance in the composite powders.

Although the composite powders were collected in an air environment, they reacted violently with distilled water at temperatures as low as 0 °C. At 30 °C, the powders achieved a hydrogen conversion yield of 91.30% within 16 minutes. In addition, the hydrogen produced by the composite powders in this study could be used directly in proton exchange membrane fuel cells (PEMFC). In a word, this work is important to provide high efficient, high oxidation resistance and low cost hydrogen generation materials for fuel cells or other hydrogen energy devices, especially in extreme environments such as the deserts, polar region, and outer space.

## Methods

Al (99.9%), Bi (99.9%) and Sn (99.99%) were used as starting materials. The Al, Bi, and Sn were firstly melted by induction melting furnace under argon atmosphere. In order to get homogeneous liquid phase, the temperature of the melting materials was kept 50~100 °C higher than the liquid miscibility gap for over 10 minutes. Then the melting materials were atomized using a high-pressure argon gas flow of approximately 5–10 MPa. The Al-based powders were collected in an air environment with a relative humidity of 40% at room temperature. During the collection process, the powders were exposed to air for about five minutes. After that, the powders were stored in plastic capsules with a vacuum of −0.1 MPa. The powders used for oxidation resistance experiment were stored in an air environment with a relative humidity of 20% at 30 °C.

The methods and devices used to conduct the hydrogen generation measurements were described in our previous study^31^. The hydrolysis reactions are performed in a 250 ml flask reactor with two openings, which is put in water bath to control the temperature of the reactants during the reaction. In each experiment, 100 ml distilled water is firstly placed in the reactor, after the water temperature achieves the setting one, 0.35 g of the Al-based powders is charged into the reactor. Hydrogen measurement starts immediately when the powders contact with the distilled water, and the volume of hydrogen is measured by the water displacement method. The generated hydrogen gas flows through a drying pipe in order to dry hydrogen gas and then the hydrogen gas is collected in a container filled with water, from which water is displaced into a flask. Both the drying pipe and the container are placed in water bath of 25 °C. The mass of the flask with water displacement by hydrogen gas is measured by electronic balance, and the data is recorded by the computer that connected with the electronic balance. In general, the density of water is 1 g/cm^3^, namely the mass of water is just the volume of generated hydrogen at 25 °C. The hydrogen conversion yield (%) was defined as the ratio of the volume of H_2_ generated by 1 g powders and the theoretical volume of H_2_ generated by 0.8 g Al at 25 °C and 1 atm (1086.4 ml).

The morphologies and microstructures of the composite powders were analyzed using scanning electron microscope (SEM) (LEO1530, LEO, Germany) and EPMA (JXA-8100R, JEOL, Japan). Energy dispersive spectrometer (EDS) was used to measure the metal content of the Al-based composite powders, as well as the compositions of different phases. The crystal structures of the Al-based powders were analyzed using an X-ray diffractometer (D/MAX-Ultima IV, RIGAKU Corporation, Japan). The solid hydrolysis products were obtained by pouring the solutions through a filter on the accessory flask with a side arm. Then the samples were dried under a vacuum at 50 °C for 24 hours.

## Additional Information

**How to cite this article**: Wang, C. *et al.* A Novel Self-Assembling Al-based Composite Powder with High Hydrogen Generation Efficiency. *Sci. Rep.* **5**, 17428; doi: 10.1038/srep17428 (2015).

## Supplementary Material

Video S1

Supplementary Information
