# Supplementary material for: A Novel Self-Assembling Al-based Composite Powder with High Hydrogen Generation Efficiency
Source: Sci Rep. 2015 Nov 30;5:17428. doi: 10.1038/srep17428 (PMC4663537; doi:10.1038/srep17428)
Supplement: Supplementary Information [file srep17428-s2.pdf]

Supplementary Information

A Novel Self-Assembling Al-based Composite Powder with High Hydrogen Generation Efficiency

Cuiping Wang[1,2,+], Yuheng Liu[1,+], Hongxin Liu[1], Tao Yang[1], Xinren Chen[1], Shuiyuan Yang[1,2], and Xingjun Liu[1,2,3,*]

[1] Department of Materials Science and Engineering, College of Materials, Xiamen University, Xiamen, 361005, P.R. China
[2] Research Center of Materials Design and Applications, Xiamen University, Xiamen, 361005, P.R. China
[3] Collaborative Innovation Center of Chemistry for Energy Materials, Xiamen University, Xiamen 361005, P. R. China
*corresponding author: lxj@xmu.edu.cn (X.J. Liu)
[+] these authors contributed equally to this work

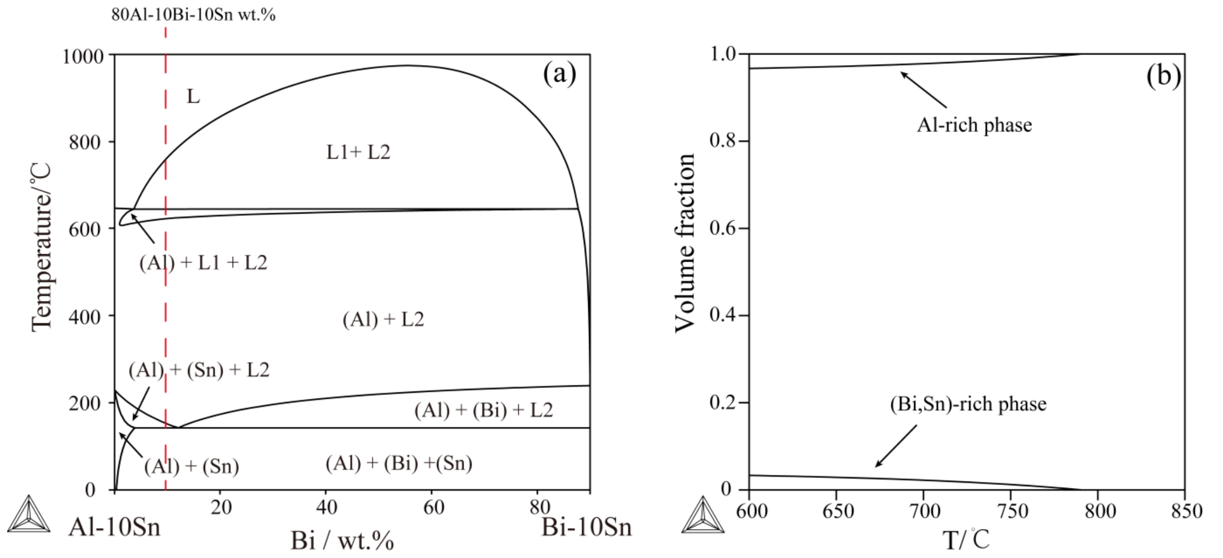

Figure S1. (a) calculated vertical section at 10 wt.% Sn of the Al-Bi-Sn system; (b) calculated volume fractions of the separated liquid phases in 80Al-10Bi-10Sn wt.% system.

As shown in Figure S1(a), a stable liquid miscibility gap exists when the powder was comprised of 80Al-10Bi-10Sn wt.%. However, Figure S1(b) displays that the volume of the (Bi, Sn)-rich phase was too small to form a shell around the entire powder surface.

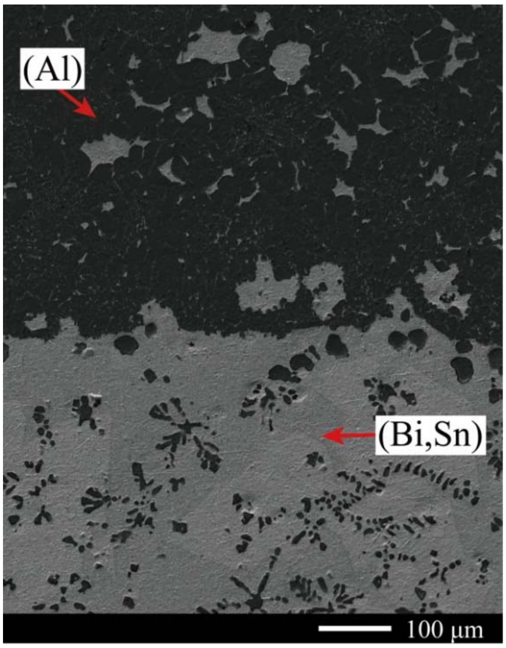

Figure S2. Back scattered electron (BSE) image of the 80Al-10Bi-10Sn wt.% bulk materials melted with a resistance furnace. The phase separations are visible in this figure.

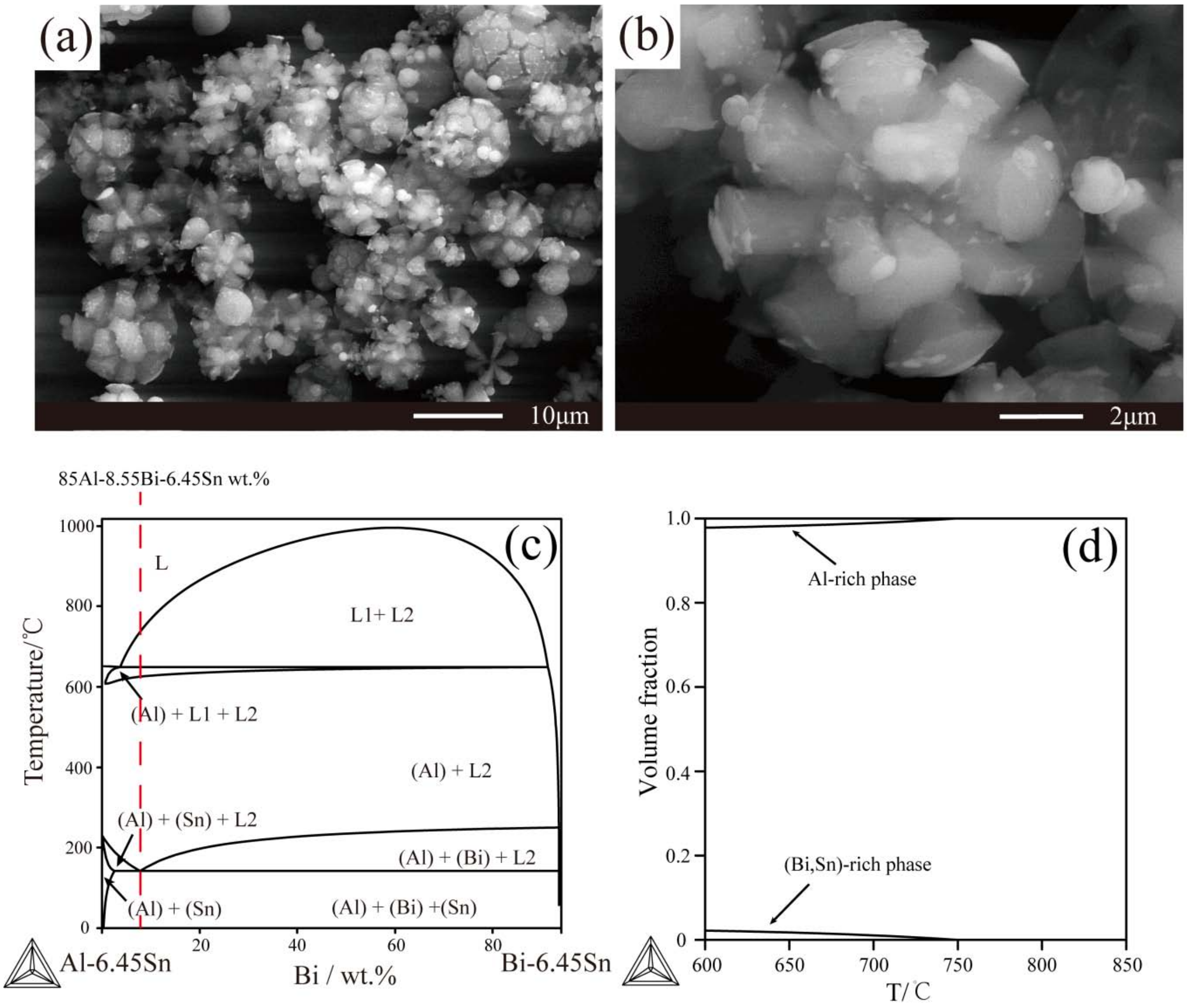

Figure S3. (a, b) SEM images of the surfaces of the 85Al-8.55Bi-6.45Sn wt.% powders; (c) calculated vertical section at 6.45 wt.% Sn of the Al-Bi-Sn system; (d) calculated volume fractions of the separated liquid phases in the 85Al-8.55Bi-6.45Sn wt.% system.

As shown in this figure, the compositions of Bi and Sn were located within the eutectic phase of the Bi-Sn system. In addition, the powder surfaces exhibited more cracks.

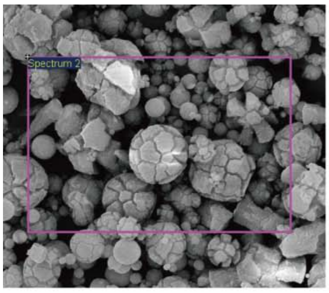

Element	Weight%	Atomic%
O K	6.66	12.96
Al K	71.56	82.57
Sn L	10.86	2.85
Bi M	10.93	1.63
Totals	100.00	

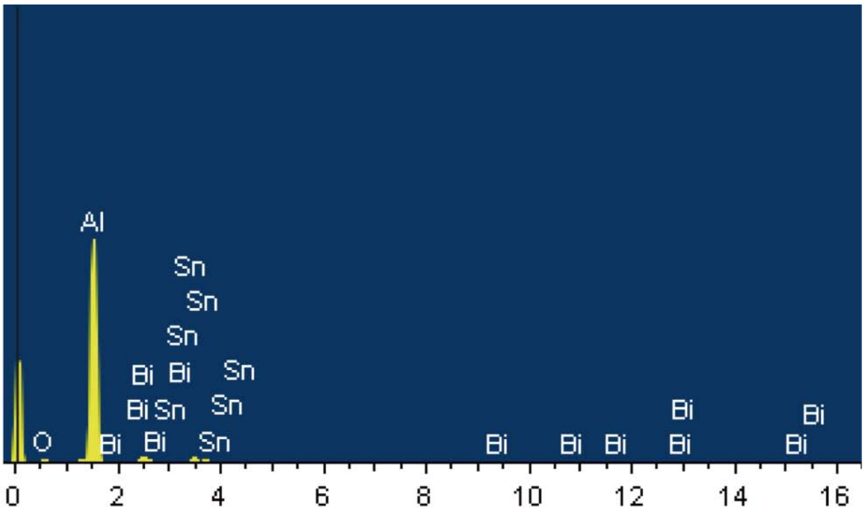

Figure S4. EDS results of the 80Al-10Bi-10Sn wt.% composite powders.

As we can see that, the composition of oxygen element is 6.66 wt.%, this is caused by the unavoidable oxidation of the bare Al-phase on the powder surface during the collection process in air environment.

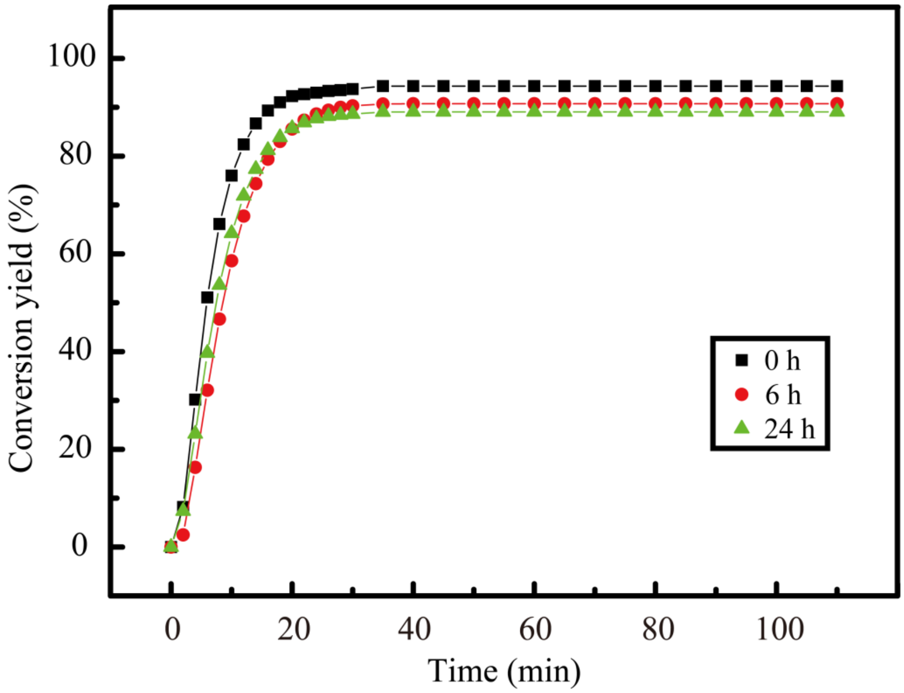

Figure S5. Hydrogen generation curves of the 80Al-10Bi-10Sn wt.% powders in distilled water at 30°C after air exposed for different time.

In order to testify the oxidation resistance of the composite powders, meanwhile, to exclude the influences of vapor, the composite powders were placed in an air environment with relative humidity of 20% at 30°C for different time and then reacted with distilled water at 30°C to produce hydrogen. The hydrogen generation curves of the powders air exposed for 6 hours and 24 hours are showed in Figure S5. As we can see that, after exposed in air for 6 hours, the conversion yield of hydrogen just slightly decreases from 92.02% to 90.72%. When the exposure time increases to 24 hours, the hydrogen conversion yield has no obvious decrease and still achieves 89.05%, indicating that the composite powders have a good oxidation resistance.

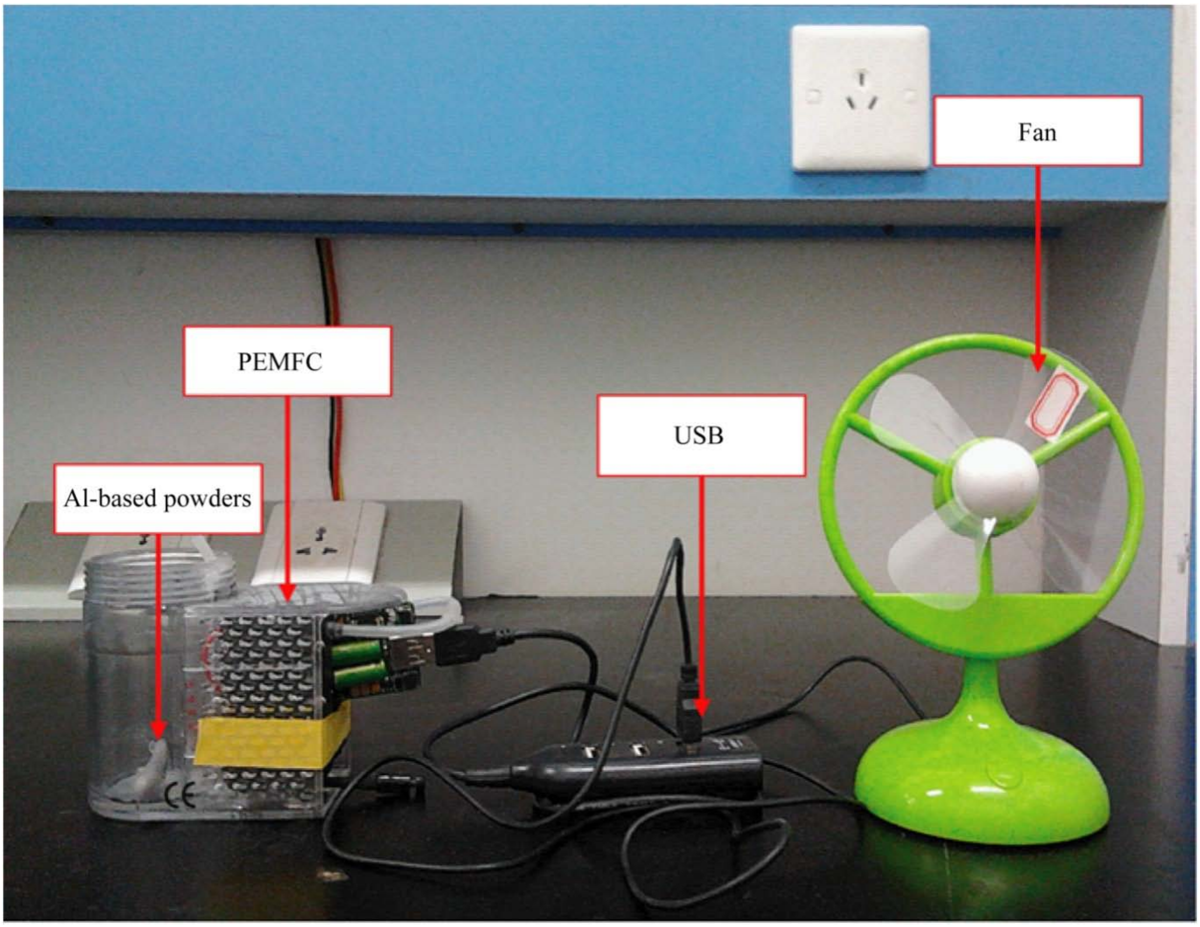

Figure S6. Hydrogen application device used in this study.

Video S1. Video demonstrating the use of the hydrogen application device used in this study.

First, the Al-based powders and distilled water were placed in the reaction chamber. Violent reactions occurred immediately. Then, the hydrogen produced by these reactions flowed through the proton exchange membrane fuel cell (PEMFC) (Zhongjing New Energy, China), where it was translated into electric energy. After the remaining air was released from the system, the red signal light flickered, indicating that the hydrogen flow was not yet stable. The blue signal indicated that the electric energy was ready for application. The LED light, fan, and mobile phone in this system could be charged using a USB interface.

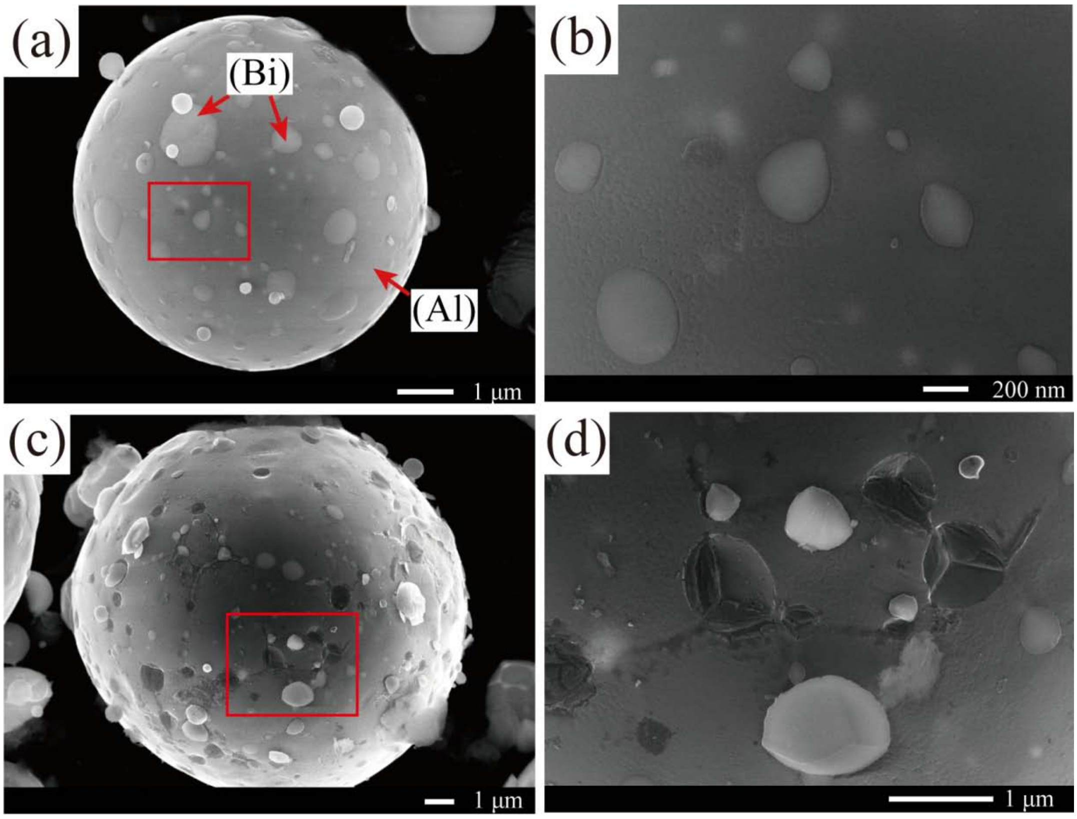

Figure S7. SEM images of the 80Al-20Bi wt.% powders reacting with distilled water at 30°C for different time: (a-b) before reaction; (c-d) 1 minute.

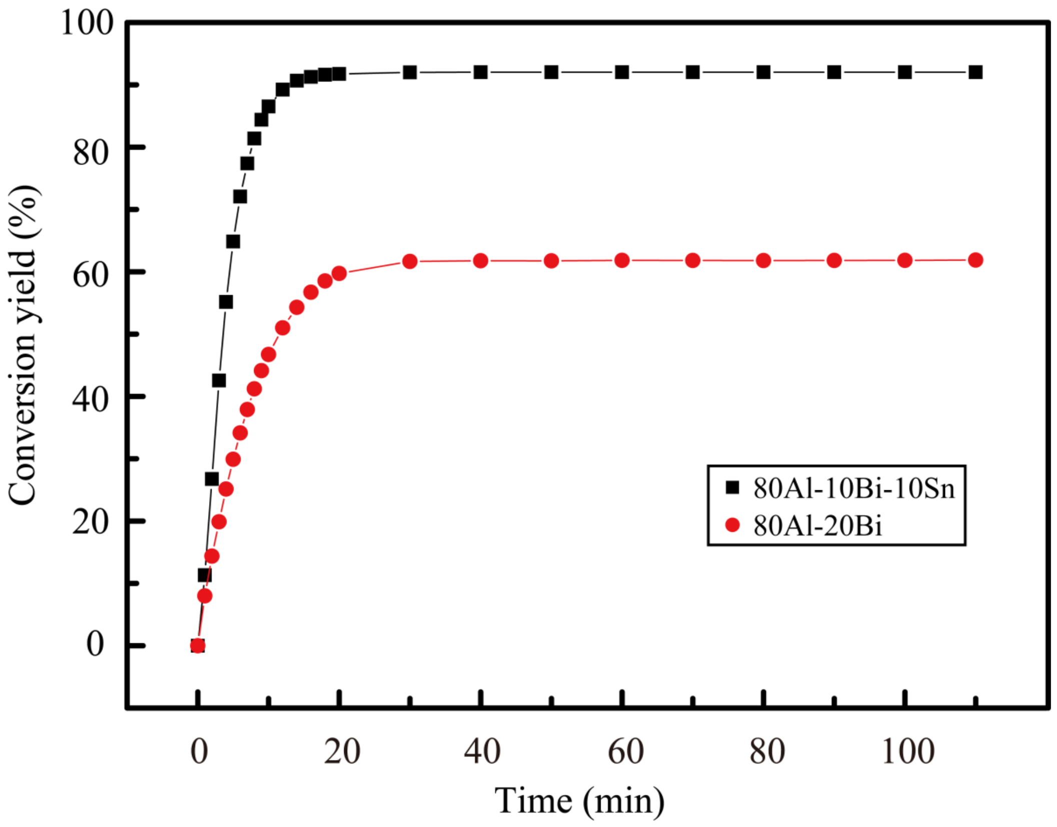

Figure S8. Hydrogen generation curves of the 80Al-20Bi wt.% and 80Al-10Bi-10Sn wt.% powders in distilled water at 30°C.

Figure S7 displays the SEM images of the 80Al-20Bi wt.% powders before and after one minute of reaction with distilled water. As shown in (a) and (b), Bi-rich phases form round spots, aggregating on the powder surface, especially on the grain boundaries. After reacting with distilled water for one minute, seeing (c) and (d), Bi-rich spots on the grain boundaries or triple junctions of the Al grains drop off. As a result, lots of fresh surfaces of Al grains are exposed to water, from where violent hydrolysis reactions start. The hydrogen generation curves of the 80Al-20Bi wt.% powders in distilled water at 30°C is shown in Figure S8. Comparing with the 80Al-20Bi wt.% powders, the 80Al-10Bi-10Sn wt.% powders show superior performances both in reaction rate and conversion yield.
